# Mucus is more than just a physical barrier for trapping oral microorganisms

**DOI:** 10.1080/20002297.2020.1788352

**Published:** 2020-07-03

**Authors:** Ingar Olsen

**Affiliations:** Department of Oral Biology, Faculty of Dentistry, University of Oslo, Oslo, Norway

**Keywords:** Mucus, mucins, glycans, *Pseudomonas aeruginosa*, streptococci, *Candida albicans*, virulence, attenuation, biofilm, communication

## Abstract

Mucus is thought to serve as a protective coating on wet epithelial surfaces. Recent research has shown that glycans, which are branched sugar molecules found in mucin, a part of mucus, can prevent bacteria from communicating with each other and forming biofilms. This could hinder microbes from causing infections. The present editorial, focusing on a paper by Wheeler et al. [1], published in October 2019 in Nature Microbiology, describes how mucus can attenuate the virulence of *Pseudomonas aeruginosa*. In addition, streptococci and *Candida albicans* can be ‘tamed’ by mucin.

Epithelia in the body, e.g. in the oral cavity, are lined with a slimy, hydrated mucus gel, which protects against invading microorganisms [1,2]. Therefore, infections under intact mucus are rarely seen. Recent research has shown that mucus is not just a lubricant or physical barrier able to trap bacteria, viruses and yeasts by acting as a first line of defense [3]. It can even prevent bacteria from attaching to surfaces, communicating with each other and forming biofilm [4]. This could make the microbes less harmless and help establish and maintain a diverse microbiome.

## Mucin ‘tames’ the virulence of *Pseudomonas aeruginosa* and other oral organisms

A recent study in Nature Microbiology [4] from Massachusetts Institute of Technology focused on the interaction between mucin-associated glycans (mucin glycans) and *Pseudomonas aeruginosa* where mucin glycans were shown to ‘tame’ the virulence of this opportunistic bacterium. *P. aeruginosa* is a facultative Gram-negative rod that is not pathogenic in healthy individuals but can cause serious infections in patients with reduced immunity, aberrant mucus production and burn wounds [5,6]. In cases with primary extra-oral *P. aeruginosa* infections, it is not unusual to find this bacterium in the oral cavity [7–10]. *P. aeruginosa* is also occasionally isolated from primary oral diseases such as periodontitis [11–13] and oral squamous carcinoma [14].

Other oral microorganisms such as streptococci and *Candida albicans* can also have their virulence attenuated by mucins [1]. Thus, salivary mucins can disperse bacteria through glycan-specific interactions. The fact that mucins can reduce microbial virulence in the mouth has been demonstrated in HIV/AIDS, oral candidiasis and dental caries [1]. In the presence of salivary mucins, the HIV infection of T-cells was diminished, hyphal production by *Candida* reduced, and biofilm formation by *Streptococcus mutans* decreased. Interestingly, in a dual-species model with *S. mutans* and *Streptococcus sanguinis*, the mucin MUC5B increased bacterial coexistence and, ultimately, bacterial diversity, by shifting competing species away from the biofilm and into the less competitive planktonic state [15]. Furthermore, Kavanaugh et al. [16] reported from *in vitro* experiments supplied with gene expression analysis that mucin biopolymers induced a new oval-shaped morphology of *C. albicans* where a range of genes related to adhesion, filamentation and biofilm formation was downregulated.

Defects in the production of mucus are often followed by infections and biofilm formation, as seen in patients where reduced salivation tends to disturb the balance of the oral microbiota [17] and dysregulation of select epithelial mucins can contribute to xerostomia [18,19].

## Mucin virulence-‘taming’ mechanisms for *P. aeruginosa*

It is important to keep in mind that mucins are the major structural components of mucus. They are polymers densely grafted with O-linked glycans that form a 3D scaffold inside mucus [4]. By using *P. aeruginosa* and a three-dimensional laboratory model of native mucus, mucus exposure was found to rapidly disintegrate biofilms [4]. In another study, mucins separated the cells in *P. aeruginosa* biofilms and dispersed them into suspension [20]. Exposure to mucus reduced the biofilm mass of a wild-type strain of *P. aeruginosa* (PAO1), not that of a flagellar mutant (∆*aflid*), indicating that mucus-mediated biofilm dispersal depends on an intact flagellum [4]. Furthermore, intestinal mucus was found to transcriptionally suppress expression of genes important for virulence, i.e. genes related to quorum sensing *(lasR*), biosynthesis of siderophores (*pvdA*) and type 3 secretion (*perV*). The downregulation of major virulence genes ‘disarmed’ *P. aeruginosa*. The fact that both native gastric and salivary mucus suppressed major genes related to virulence indicated that the suppression of virulence was conserved across different mucosal surfaces. When purified mucins were added back into depleted mucus, the biofilm dispersal and suppression of virulence genes were restored. Accordingly, mucus possesses factors that modulate the behaviors of bacteria both at the level of gene expression and phenotype. When native intestinal mucus solutions dispersed biofilm into the planktonic state, 70% of the cells dissociated from the biofilm surface [4]. This was due to phenotype regulation by which the mucus stimulated an active motility-driven escape. Native mucus solutions also suppressed major virulence traits, while depletion of mucous components prevented biofilm dispersal. This was partially restored when mucus filtrates were supplemented with the purified mucin MUC2. Furthermore, the depletion of gastric mucus components increased the expression of virulence genes.

The regulatory function of mucin did not alone result from the polymeric mucin structure. It was noteworthy that free mucin glycans, isolated from the mucin backbone, regulated the bacterial phenotypes, even at fairly low concentrations [4]. In whole mucus, the primary bioactive component was larger than 100 kDa. This indicated that mucin polymers could be candidates. It was also plausible that the regulatory function of mucins depended upon glycan complexity because monosaccharides did not reduce the virulence of *P. aeruginosa*. Therefore, mucin-associated glycans might be potent host signals for ‘taming’ of microorganisms and making them less harmful.

To examine whether mucins triggered a global transcription response, RNA sequencing was performed of *P. aeruginosa* grown with or without MUC5AC or MUC5B [4]. These are the most abundant gel-forming mucins secreted in niches colonized by *P. aeruginosa* and can be found in the oral cavity, respiratory tract, eyes and middle ear. A genome-wide response was triggered by both mucins. A number of virulence pathways were suppressed, including type-1, −2, −3 and −6 secretion systems, siderophore biosynthesis and quorum sensing. Thus, protease activity and siderophore production were lower in supernatants of *P. aeruginosa* exposed to mucin than in those, which had not been exposed.

Also, adhesion of *P. aeruginosa* to glass, plastic and live human epithelial cells (HT-29) was reduced by MUC5AC. Mucin probably suppressed the antagonistic interaction between *P. aeruginosa* and epithelial cells exerted by its toxins. *P. aeruginosa*-mediated epithelial cell death took place in a dose-dependent manner.

Some genes were also upregulated, such as genes in the denitrification pathways [4]. Certain metabolic genes were differentially regulated, e.g. those related to fumarate metabolism and amino acid and C5-carboxylate transport. Such metabolic changes often correlate with changes in virulence. There was no increase in virulence pathways among upregulated genes, but there was an indication that mucin suppressed the expression of virulence genes across a range of experimental conditions.

To test the effect of mucins in a system with multiple cell types and intact immune functions, porcine burn wounds were infected with *P. aeruginosa* [4]. Exposure to MUC5AC in a wound dressing had caused a two-log reduction in colony-forming units (CFUs) of *P. aeruginosa* one week after infection. There was no reduction after mucin-free mock treatment. The bacterial clearance was probably not due to direct killing by MUC5AC since bacterial viability was not reduced by isolated mucins. The effect of mucin was not a result of changes in phenotypic features such as flagellar motility or ability to aggregate bacterial cells, but rather due to a parallel effect yielding phenotypic switches while maintaining the attenuating effect on virulence genes. Interestingly, the polymeric structure of mucin was not sufficient for establishing the virulence-attenuating effects. It was therefore speculated that a specific biochemistry present in mucins was necessary to reduce the virulence of *P. aeruginosa*.

Native mucins contain a large number of complex glycan structures covalently linked to serine and threonine [21–23]. This creates a wealth of possibilities for affecting gene expression. To establish whether glycans contributed to the neutralizing effect of mucins, glycans isolated from the MUC5AC protein backbone by alkaline β-elimination was tested [4]. Functional enrichment analyses confirmed that the same virulence pathways suppressed by intact MUC5AC were suppressed as by free MUC5AC glycans. Accordingly, an integral part of mucin could be the primary virulence neutralizing factor in mucus rather than changes in motility or aggregation. Monosaccharides present in the mucin glycans did not suppress the production of virulence factors. Therefore, the complex arrangement and stereochemistry of these sugar residues were probably critical for their function as regulatory signals.

## Remaining questions

One remaining question is how mucin glycans react with and are recognized by *P. aeruginosa* at the molecular level. Wheeler et al. [4] suggested that glycans may directly serve as a signal by using a carbohydrate binding site in a global regulatory system, e.g. that affecting the secondary messenger c-di-GMP or the non-coding RNAs rsmY and rsmZ [24,25]. Another possibility is that glycan may stimulate metabolic changes by serving as a substrate for nutrition or by regulating signaling pathways by interactions with specific *P. aeruginosa* lectins or surface adhesins. Wheeler et al. [4] speculated that the structural diversity of mucin-associated glycans enabled them to interact with several bacterial receptors and mediate distinct functions. These notions will have to await further research. Nevertheless, Wheeler et al.'s [4] findings have demonstrated previously unrecognized roles for mucin glycans to serve as potent host-derived regulators of the bacterial phenotype. This could prevent mucosal infections while at the same time establish and preserve a diverse microbiota.

Why diseased mucus no longer maintains the ability to attenuate virulence but promote biofilm formation and antibiotic resistance, as reported by Landry et al. [26] and Secor et al. [27], is another open question. Wheeler et al. [4] suggested that alterations to mucin glycosylation patterns in disease, e.g. increased sialylation, could change the binding properties of mucin with microorganisms and its protective function. By establishing a mucin-glycan library for the hundreds of different glycans present in mucus, the regulatory code might be solved by identifying specific bioactive glycans. These might serve as therapeutics for the treatment of intractable bacterial infections and the stabilization of the healthy microbiota.

Further research should also be undertaken to see if (1) receptors on the microbe cell surfaces can interact with glycans, (2) glycans can block horizontal gene transfer preventing the spread of drug resistance, (3) artificial mucus can be developed to treat infections where mucus production is suppressed and (4) mucus-associated glycans can have sugars able to regulate microbial behavior, thereby creating and maintaining a diverse microbiota. In addition, the microbial effect of mucus needs to be elucidated on a much wider spectrum of microorganisms in the oral cavity. This is particularly important because it has been shown that gut bacteria such as *Clostridiales* members utilize intestinal mucins and the mucin utilizer *Peptostreptococcus russellii* reduces susceptibility to epithelial injury in mice, probably by producing the tryptophan metabolite indoleacrylic acid, which suppresses inflammation [28].
